# Review of key knowledge gaps in glucose-6-phosphate dehydrogenase deficiency detection with regard to the safe clinical deployment of 8-aminoquinoline treatment regimens: a workshop report

**DOI:** 10.1186/1475-2875-12-112

**Published:** 2013-03-27

**Authors:** Lorenz von Seidlein, Sarah Auburn, Fe Espino, Dennis Shanks, Qin Cheng, James McCarthy, Kevin Baird, Catherine Moyes, Rosalind Howes, Didier Ménard, Germana Bancone, Ari Winasti-Satyahraha, Lasse S Vestergaard, Justin Green, Gonzalo Domingo, Shunmay Yeung, Ric Price

**Affiliations:** 1 Global and Tropical Health Division, Menzies School of Health Research, Charles Darwin University, Darwin, NT, Australia; 2 Research Institute for Tropical Medicine, Manila, Philippines; 3 Australian Army Malaria Institute, Enoggera, Australia; 4 Queensland Institute of Medical Research, University of Queensland, 300 Herston Road, Herston, Brisbane, Queensland, 4006, Australia; 5 Eijkman-Oxford Clinical Research Unit, Jakarta, Indonesia; 6 Spatial Ecology and Epidemiology Group, Tinbergen Building, Department of Zoology, University of Oxford, Oxford, UK; 7 Institut Pasteur of Cambodia, Phnom Penh, Cambodia; 8 Shoklo Malaria Research Unit, Mae Sot, Thailand; 9 WHO/WPRO, Manila, Philippines; 10 GlaxoSmithKline, Stockley Park, Uxbridge, UK; 11 PATH, Seattle, SE, USA; 12 The Mahidol Oxford Tropical Medicine Research Unit, Bangkok, Thailand; 13 Centre for Tropical Medicine, Nuffield Department of Medicine, University of Oxford, Oxford, UK

**Keywords:** Malaria, Vivax, Falciparum, 8-aminoquinolines, Primaquine, Tafenoquine, G6PD, Deficiency, Diagnostic tests

## Abstract

The diagnosis and management of glucose-6-phosphate dehydrogenase (G6PD) deficiency is a crucial aspect in the current phases of malaria control and elimination, which will require the wider use of 8-aminoquinolines for both reducing *Plasmodium falciparum* transmission and achieving the radical cure of *Plasmodium vivax*. 8-aminoquinolines, such as primaquine, can induce severe haemolysis in G6PD-deficient individuals, potentially creating significant morbidity and undermining confidence in 8-aminoquinoline prescription. On the other hand, erring on the side of safety and excluding large numbers of people with unconfirmed G6PD deficiency from treatment with 8-aminoquinolines will diminish the impact of these drugs. Estimating the remaining G6PD enzyme activity is the most direct, accessible, and reliable assessment of the phenotype and remains the gold standard for the diagnosis of patients who could be harmed by the administration of primaquine. Genotyping seems an unambiguous technique, but its use is limited by cost and the large range of recognized G6PD genotypes. A number of enzyme activity assays diagnose G6PD deficiency, but they require a cold chain, specialized equipment, and laboratory skills. These assays are impractical for care delivery where most patients with malaria live. Improvements to the diagnosis of G6PD deficiency are required for the broader and safer use of 8-aminoquinolines to kill hypnozoites, while lower doses of primaquine may be safely used to kill gametocytes without testing. The discussions and conclusions of a workshop conducted in Incheon, Korea in May 2012 to review key knowledge gaps in G6PD deficiency are reported here.

## Background

The 8-aminoquinolines, a class of drugs discovered almost 80 years ago, remains unique among anti-malarial drugs in having activity against the sexual stages (gametocytes) of *P. falciparum* and dormant stages of *Plasmodium vivax* (hypnozoites). Despite being an intrinsic part of most national and international malaria treatment guidelines, the use of primaquine in the treatment of *P. vivax* as well as *P. falciparum* is often deferred by attending physicians and healthcare providers. Lingering uncertainty regarding the efficacy and tolerability of primaquine is the most likely reason for this under-utilization. Yet the unique features of primaquine could be critical to tackle three of the most important challenges to malaria therapeutics: 1) The interruption of malaria transmission: despite massive gains made in reducing the burden of malaria in Asia as well as Africa, interruption of the residual transmission has been challenging. Expanding the use of primaquine has been proposed to reduce the transmission potential in both symptomatic patients and asymptomatic parasitaemia. A World Health Organization (WHO) expert committee came to the conclusion that primaquine can be safely used as a single dose (0.25 mg base/kg) even in individuals with G6PD deficiency [1]. Evidence including series of studies from China suggests that such a single low-dose primaquine may be sufficient to kill gametocytes [2,3]. 2) Reducing the spread of artemisinin resistant *P. falciparum* strains: one of the greatest threats to current treatment strategies is the emergence and spread of *P. falciparum* strains resistant to artemisinin derivatives [1-3]. Artemisinin resistance was first reported in Western Cambodia and has been documented on the western border of Thailand by 2012 [4-6]. Several approaches using gametocytocidal drugs to interrupt malaria transmission and ultimately eliminate malaria have been proposed. In order of increasing complexity these interventions include adding gametocytocidal drugs as part of case management, screening followed by treatment of cases, and mass drug administrations. 3) The radical cure of *P. vivax*: although current malaria control efforts have been successful in reducing the transmission of falciparum malaria they have had a more limited effect on the transmission of vivax malaria, resulting in a relative increase of the proportion of malaria cases due to *P. vivax* in areas where effective malaria control programmes have been implemented [7]. This reflects the different transmission dynamics of *P. vivax* infections and the difficulties in eliminating the dormant liver stages. To address this problem malaria control programmes need to be able to deploy effective radical cure of *P. vivax*, to kill both erythrocytic and exo-erythrocytic stages of the parasite. The only drugs currently available with activity against all stages of *P. falciparum* gametocytes as well as *P. vivax* hypnozoites are the 8-aminoquinoline drugs, of which only primaquine and the prodrug bulaquine are currently licensed. Bulaquine is produced by Central Drug Research Institute (CDRI), Lucknow and licensed in India only [8,9]. A long-acting 8-aminoquinoline, tafenoquine, has been in development for nearly 30 years and could be licensed in years to come.

Despite the therapeutic advantages of the 8-aminoquinolines, the wider use of drugs such as primaquine is restricted by their toxicity profile. The most important adverse effect of 8-aminoquinolines is dose-related haemolysis. In people with an inherited deficiency of glucose-6-phosphate dehydrogenase (G6PD) a single dose of primaquine can trigger a severe haemolysis reaction. The first report of 8-aminoquinolines-related haemolysis was recorded in 1926 following the administration of pamaquine, the earliest available 8-aminoquinoline, also known as plasmoquine or plasmochin: “On the fourth day of treatment, after the fever had disappeared the patient developed profound anaemia, leucocytosis, jaundice, nausea, vomiting, and somnolence. He died within 48 hours of the onset of this sudden attack. The toxic influence of plasmochin compound was suspected to have played an important role in the cause of death” [10]. It took until the 1950s for researchers to discover variations in G6PD which confer a risk for haemolysis following the administration of primaquine [11,12]. The precise mechanism of haemolysis remains unknown. G6PD catalyses the rate-limiting reaction of the hexose monophosphate shunt, the primary source of electrons to reduce nicotinamide adenine dinucleotide phosphate (NADP+). NADPH is key to maintaining the redox equilibrium within erythrocytes (red blood cells, RBC). It has been proposed that an oxidized metabolite of primaquine may precipitate the molecular events leading to haemolysis. The appearance of Heinz bodies, potentially derived from haem moieties dislodged from their hydrophobic globin folds, signal the onset of haemolysis.

Haemolysis in individuals with G6PD deficiency has been reported to follow therapy with a range of anti-malarial drugs (8-aminoquinolines, including primaquine, tafenoquine and pamaquine), sulphones (dapsone), sulphonamides (such as sulphanilamide, sulphamethoxazole, and mafenide), analgesics (such as aspirin, phenazopyridine, and acetanilide), non-sulpha antibiotics (nalidixic acid, nitrofurantoin, isoniazid, and furazolidone), methylene blue and naphthalene [13]. In addition, haemolysis can also be induced by foods (such as fava beans), henna, and infections (including Hepatitis viruses A or B, cytomegalovirus, pneumonia, and typhoid fever) [13,14]. Keeping in mind that the clinical evidence linking some of the compounds with haemolysis in G6PD-deficient people is weak, there may be no causal relationship between the compound and the less frequent reactions.

Following administration of a standard 14-day course of primaquine (0.25 mg/kg body weight per day) the majority of treated people experience some degree of subclinical haemolysis regardless of G6PD status [15]. The clinical significance of a haemolytic episode can range from mild and self-limiting to severe and life threatening, depending upon the exposure to the drug, the specific variant of G6PD deficiency and incompletely understood host factors. Unfortunately, primaquine sensitivity among the many G6PD variants has only been characterized reliably for two variants, Mediterranean and Mahidol. These variants represent severe and moderate degrees of sensitivity to primaquine respectively. African A-, frequently considered a variant associated with mild disease, can also result in more severe illness following primaquine administration [16,17]. Therefore, the risk of serious harm in any given population exposed to primaquine therapy remains largely unknown. There is a possibility that other variants of extreme sensitivity to primaquine, similar to the Mediterranean variant, also exist, but are as yet undescribed. A small fraction of people receiving primaquine will haemolyse sufficiently to have clinical signs, including clinical anaemia, bilirubinaemia resulting in jaundice, and darkly coloured urine. Severe haemolysis can lead to serious clinical consequences including renal failure and haemodynamic compromise [18]. Potentially life-saving procedures such as blood transfusions and renal dialysis are expensive, carry a risk of adverse events on their own, and are often not readily available in poorly resourced communities at high risk for malaria.

There is little if any information on the safety of primaquine use during pregnancy. Since the unborn child is not accessible to testing there is a risk of exposing a G6PD deficient child to primaquine which at least in theory could result in hydrops fetalis. Women who perceive themselves at risk of being pregnant should not be treated with primaquine. There is a need for pharmacovigilance when primaquine is used in populations which could include pregnant women [19].

The incidence of severe adverse reactions secondary to primaquine administration is difficult to determine since most reports do not provide certainty regarding the numerators or the denominators involved. A major review of the safety data related to primaquine and its predecessors plasmoquine or plasmochin archived by the WHO and the League of Nations detected 13 deaths associated the administration of primaquine. Acknowledging the methodological limitations in estimating the denominator the investigators calculated “the risk of death associated with primaquine ingestion at approximately 1 in 692,307” [20]. The risk of severe adverse events was related to the total dose of primaquine and the age of the patient. The investigators estimated that the risk of severe haemolysis in mass administrations of primaquine was 1.8 per million.

Uncertain about the potential risks of primaquine administration, policymakers and practitioners are often reluctant to put patients at risk of the potential consequences of primaquine toxicity. Few patients will blame their healthcare provider for a malaria relapse months after treatment in contrast to an episode of severe haemolysis immediately following the start of treatment. From the practitioner’s perspective it may appear advantageous to withhold primaquine therapy, particularly if patients are not requesting it.

Primaquine toxicity in patients with G6PD deficiency is a significant barrier to the effective delivery of therapies essential to combating the endemic malarias. A more satisfactory approach to the detection and management of malaria patients with G6PD deficiency would unlock a much broader anti-malarial armamentarium. This review discusses the key gaps in understanding the G6PD deficiency problem and provides strategies for its detection and management.

### Genetics of G6PD deficiency

The gene encoding the G6PD enzyme is located at the q28 locus on the X-chromosome. As a result, the phenotype due to genetic mutations is more likely to be manifest in males. Heterozygous females display varying levels of enzyme deficiency due to the partial inactivation of one female X chromosome a phenomenon first described by Mary Lyon in 1961 and hence known as lyonisation [21]. Homozygous deficient females are rare due to the relative improbability of such inheritance. The WHO groups G6PD genetic variants somewhat arbitrarily into five classes based on levels of enzyme activity. According to this WHO classification thresholds of 10 and 60% are used to define the degree of G6PD activity [22].

I. Severe deficiency (<10% activity) with chronic (non-spherocytic) haemolytic anaemia;

II. Severe deficiency (<10% activity), with intermittent haemolysis;

III. Mild deficiency (10-60% activity), haemolysis with stressors only;

IV. Non-deficient variant (60 - 100% activity), no clinical sequelae;

V. Increased enzyme activity (>100% activity), no clinical sequelae.

With the exception of rare sporadic WHO class I individuals, most G6PD-deficient individuals are asymptomatic. One hundred and eighty-six genetic determinants of G6PD deficiency have been described, including point mutations, deletions, and insertions [14,23]. Different polymorphisms result in a spectrum of enzyme deficiencies ranging from no, mild to severe effects. The combination of allelic variations at positions 202 and 376 of the G6PD gene has been investigated extensively in sub-Saharan Africa [24]. Relative to the wild-type G6PD B haplotype (202G/376A), the A haplotype (202A/376A) results in 85% enzyme activity, whilst the A haplotype (202A/376G) results in a more severe phenotype with just 12% of mean enzyme activity [25]. In most regions with high G6PD prevalence several polymorphic alleles are in circulation with the notable exception of sub-Saharan Africa where A is thought to cause 90% or more of G6PD deficiency [14]. Other relatively common polymorphisms, e.g., G6PD-Mediterranean, G6PD-Canton, or G6PD-Mahidol are associated with class II or class III deficiencies in the WHO classification. Considering the overall paucity of data, more recent reports question the relevance of the classification especially for policymakers [16].

### The geographic distribution of G6PD deficiency

**Figure 1:**
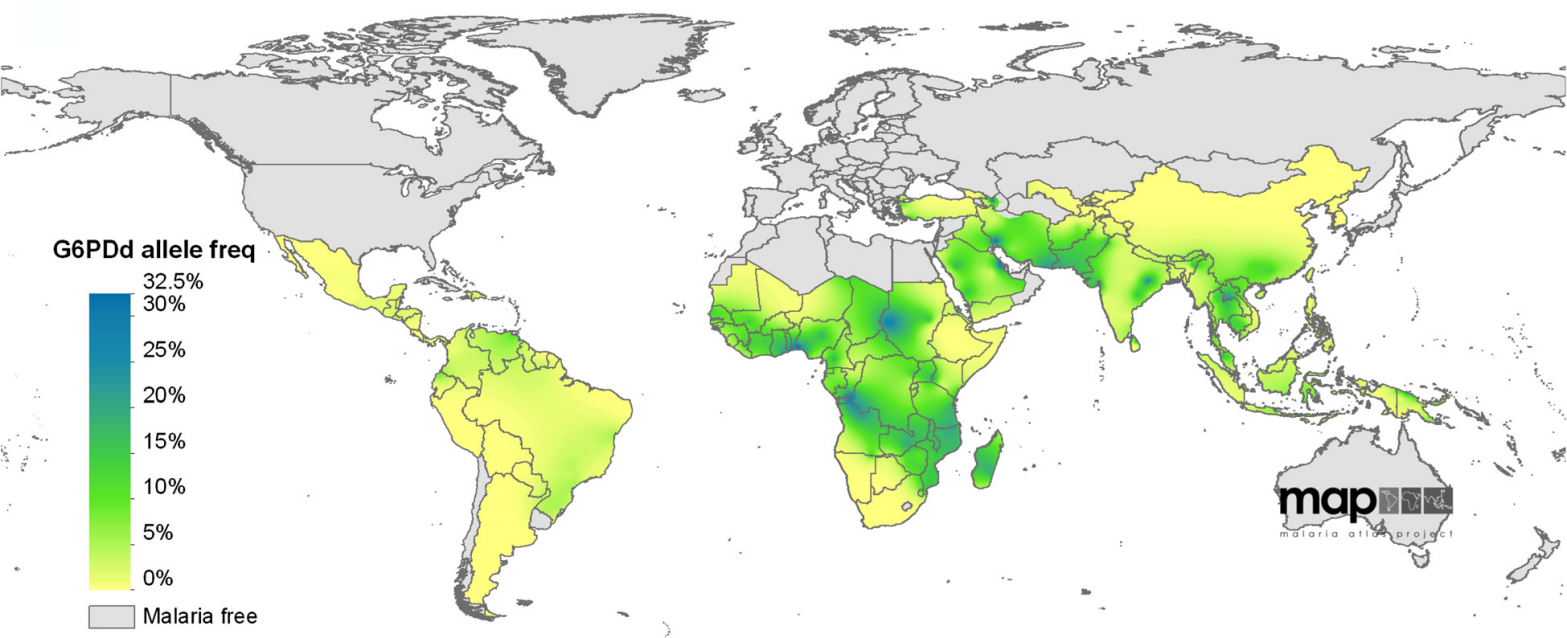
**The world map **[30]** of G6PD alleles associated with enzyme deficiency.**

It has been estimated that more than 400 million people worldwide are affected by G6PD deficiency [14]. Individuals with G6PD deficiency have an evolutionary survival advantage in malaria-endemic regions as G6PD deficiency confers a degree of protection against severe malaria [26,27]. The selective advantage of G6PD deficiency under malaria pressure has left its mark in the human genome as a “selective sweep” surrounding the G6PD gene [28,29]. Not surprisingly G6PD deficiency is most prevalent in regions where malaria is or was prevalent (Figure 1).

Howes and co-workers have identified 1,734 community G6PD surveys globally of which 1,289 (74%) were conducted in malaria-endemic countries and used this evidence-base to model a continuous prevalence map of the deficiency [30]. The prevalence of G6PD deficiency was lowest or absent in the Americas, and highest in sub-Saharan Africa. The region with the single highest predicted prevalence was in the eastern province of Saudi Arabia. The prevalence of G6PD deficiency tended to be lower, but heterogeneous across central and Southeast Asia, with “hotspots” identified in eastern India, at the Thai-Lao border and in the Solomon Islands. Despite the very large number of studies under review there remains uncertainty regarding the geographic distribution of G6PD deficiency prevalence in many areas. Uncertainty metrics accompanying the modelled map allow prioritization of areas where additional community surveys are needed. Furthermore, the prevalence of the deficiency is far from homogeneous, with considerable variations even within countries, further emphasizing the need for additional data. An acknowledged limitation of the currently available information is the variability in G6PD deficiency tests used. It is difficult to extrapolate from the reported prevalence of G6PD deficiency to the risk of severe primaquine-induced haemolysis. The modelled prevalence maps are supplemented with data on genetic variants, which allows a better prediction of the risk of haemolysis. The maps will hopefully help policymakers to optimize safer strategies to deploy primaquine.

### Tests to detect G6PD deficiency

The available tests to assess an individual’s G6PD activity status can be broadly divided into genetic and phenotypic/biochemical approaches (see Table 1).

**Table 1:** Summary phenotypic tests

	**Description**	**Setting required**	**Reference**
**Direct tests**			
Spectrophotometry	Quantitative gross enzymatic activity assay. The initial reaction velocity of the G6PD reaction is measured spectrophotometrically as an increase in absorbance at 340 nm. Despite standardized methods variations in results have been observed between sites.	Requires a biochemistry laboratory and may vary with ambient temperature and humidity.	[31-34]
”Beutler’s” fluorescent spot test	A popular screening test in which a drop of blood incubated with G6PD reaction substrates is placed on filter paper and illuminated with UV light, the presence or absence of fluorescence provides a categorical measure of G6PD activity.	Recommended as the most suitable method for screening in the field, despite the need for an UV lamp, water bath incubator, and micropipette [34].	[35]
**Indirect tests**			
Methaemoglobin reduction test (MRT)	G6PD activity is assessed by first treating RBCs with nitrite and then examining the rate of NADPH-dependent methaemoglobin reduction in the presence of an appropriate redox catalyst and substrate (glucose).	Requires a biochemistry laboratory.	[36,37]
Brilliant cresyl blue, resazurin, or formazan ring tests	Indirect assays of G6PD activity translate NADPH production into a colorimetric readout using chromophores. It takes several hours to process these assays.	Requires a biochemistry laboratory.	[35,38-41]
**Cytochemical typing**			
Methaemoglobin elution test	RBCs are labelled according to their relative methaemoglobin content based on MRT.	Requires a biochemistry laboratory.	[42]
Cytofluorometric assay	Quenching of glutaraldehyde-induced autofluorescence by formazan is detected by cytofluometry. Cytofluorometric assays can provide valuable data based on individual cells, which neither genotyping nor biochemical assays can provide but require considerable resources.	Requires a laboratory with experience in cytofluometry.	[43,44]
**Rapid tests**			
Hirono – 1-methoxy PMS Sephadex method	Substrate mixture (G6P, NADP, saponine) and MTT-PMS mixture are dissolved in water and mixed with Sephadex gel.	Requires a biochemistry laboratory and requires a skilled technician.	[45]
WST8/1-methoxy PMS method	An enzymatic method which utilizes a tetrazolium salt WST8, and a PMS hydrogen carrier, 1-methoxy PMS.	The quantitative, colorimetric nature, the reduced light sensitivity and the possibility of using this method with dried bloodspots make it more suitable for field use than the Hirono method.	[38,46]
**Rapid, point-of-care tests**			
BinaxNow^®^ G6PD test	A qualitative enzyme chromatographic test distinguishes accurately between samples with G6PD activity less than 4.0 U/g of haemoglobin and those with greater enzyme activity. Approved by the United States Federal Drug Administration and rather costly. Requires an operating temperature 18° to 25°C, a cold chain for reagents and pipettes. Sensitivity 98%, Specificity 98%.	Specifically developed as a point of care test.	[47]
CareStart^®^ G6PD deficiency screening test	A qualitative enzyme chromatographic test, distinguishes between samples with G6PD activity less than 2.7 U/g of haemoglobin and those with greater enzyme activity. In development, not yet commercially available. Sensitivity 68%, Specificity 100%.	Is being developed as a point of care test and may become useful as a public health tool.	[46,48]

### DNA-based genotyping

DNA-based genotyping and targeted re-sequencing efforts have identified a range of variants associated with G6PD deficiency worldwide, including regional variants. These approaches, whilst currently not practical for point-of-care use, may facilitate large-scale screening efforts with potentially quantitative measures of deficiency. Not all G6PD variants necessarily underlie deficiency and, thus, the clinical relevance of variants needs to be assessed with phenotypic correlates of enzyme activity and the potential for 8-aminoquinoline induced haemolysis. The size (~16.2 Kb) and complexity of the G6PD gene makes comprehensive re-sequencing challenging. However, new high-throughput genotyping platforms enable rapid and cost-effective screening of predefined variants in hundreds of individuals [49]. Genotyping known polymorphisms is conceptually attractive because it provides an unequivocal answer whether a particular polymorphism is present or not. However, unknown variants, and complex haplotypic determinants may be missed. Although specificity is high, the sensitivity of genotyping approaches to detect populations at risk will vary according to the panel of genetic variants and their local prevalence. In Burkina Faso where the G6PD-A haplotype encompasses more than 90% of mutations, a genotyping assay has been successfully employed with sensitivity and specificity comparable to a phenotypic assay [50]. A more recent multicentre study conducted in Burkina Faso, Ghana, Kenya, Nigeria, Tanzania, Mali found the correlation between genotype and phenotype was in contrast rather disappointing. Phenotype test specificity in detecting hemizygous males was 71% (70/99) and 48% (12/25) for homozygous females [51]. Heterozygous females displayed a wide range of G6PD activity. Furthermore information regarding the correlation between genotyping results and clinical outcomes is very limited. The screening for single nuclear polymorphisms can be challenging in malaria endemic settings with limited resources. Screening for multiple polymorphisms and analysing the data is feasible in research settings but becomes increasingly complex. The difficulties of performing and analysing such tests in a field setting not to mention the costs are a major obstacle for the widespread use of such molecular diagnostics in field settings.

### Phenotypic testing

Phenotypic testing of the enzymatic activity of G6PD on freshly collected venous blood remains the most widely used diagnostic method and the most informative indicator of the level of enzyme function [46]. There are more than 30 G6PD phenotype deficiency-testing products currently on the market, including both quantitative and qualitative methods, however rigorous data evaluating the tests are limited. Most available assays have not been evaluated in malaria-endemic settings. Currently only two tests may be appropriate for point-of-care use: BinaxNow**^®^** and CareStart**^®^**. Phenotypic tests can be divided into four broad categories which are summarised in Table 1:

**Figure 2:**
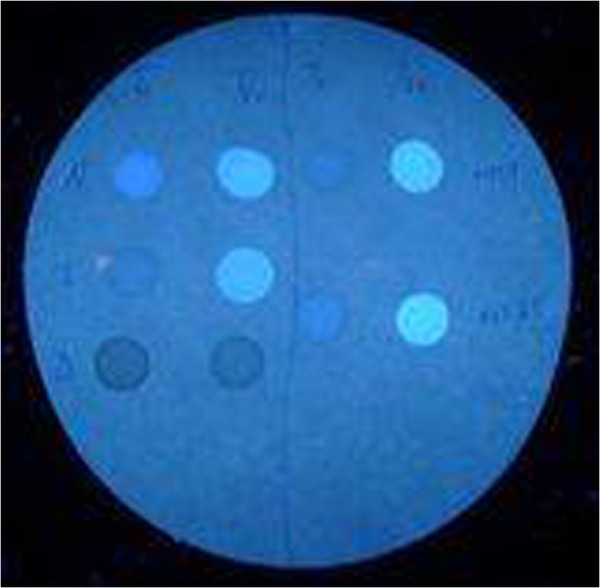
**An example of “Beutler’s” fluorescent spot test. **Samples with enzyme activity are fluorescent while samples with reduced enzyme activity produce no fluorescence.

a) **Direct tests** measure the enzymatic activity of G6PD. The standard measurement of such enzymatic activity remains spectrophotometry [31]. The basic assay of the enzyme haemolysate was first described in 1952 [32]. Commercial test kits have simplified the assays [33], but they still require a biochemistry laboratory and a skilled technician.” Beutler’s” fluorescent spot test is a popular screening test in which haemolysate is incubated with G6PD reaction substrates is placed on filter paper and illuminated with UV light (450 nm) [34]. The presence or absence of fluorescence provides a categorical measure of G6PD activity. The fluorescent spot test is currently recommended as the most suitable method for screening in the field even though it is far from ideal [34]. Besides requiring an UV lamp, water-bath incubator, and micropipettor especially in field settings it can be difficult to decide whether or not fluorescence is present. Even under ideal conditions, intermediate enzyme activity may be difficult to appropriately classify as deficient or normal (Figure 2).

b) **Indirect phenotypic tests** include the methaemoglobin reduction test (MRT) [36]. After treating RBCs with nitrite and a glucose substrate, the MRT measures the rate of NADPH-dependent methaemoglobin reduction in the presence of an appropriate redox catalyst. The rate of NADPH-dependent methaemoglobin reduction correlates with G6PD activity. Other indirect assays of G6PD activity use chromophores, such as brilliant cresyl blue, resazurin, formazan to monitor NADPH production. These tests produce a colorimetric readout.

c) **Cytochemical assays** assess the G6PD status of individual erythrocytes and hence can be used to detect hemizygous-deficient males, homozygous-deficient females, and heterozygous-deficient females [52]. Cytochemical typing methods include the older methaemoglobin elution test in which RBCs are labelled according to their relative methaemoglobin content based on the indirect MRT described above. More recently developed cytofluorometric assays detect the quenching of glutaraldehyde-induced autofluorescence by formazan using flow cytometry [43,44]. **Cytofluorometric assays** can provide valuable data based on individual cells which neither genotyping nor biochemical assays can provide. In practice such methods are limited by the need for extensive laboratory capacity including expensive equipment and skilled staff. These tests are useful in well-equipped laboratories, but are of limited use in field settings.

d) **Rapid, point-of-care tests** allow healthcare providers to diagnose G6PD deficiency in the clinical setting and could influence the case management of vivax malaria. Several tests have been developed; the more recent products approach the ease of the chromatographic malaria tests, which have revolutionized the diagnosis of malaria.

The Hirono–1-methoxy PMS Sephadex method requires a substrate mixture (G6P, NADP, saponine) and a mixture of the tetrazolium salt MTT with the phenazine methosulfate (PMS) which are dissolved in water and mixed in a Sephadex gel [45]. While this method has been used in the field the level of sophistication required to process this assay is far from trivial. The WST-8/1-methoxy PMS method (Dojindo^®^, Japan) is an improvement on Hirono’s method. This is an enzymatic method which utilizes a tetrazolium salt WST8 and a PMS hydrogen carrier, 1-methoxy PMS [38]. Specifically, the reduced light sensitivity, quantitative, colorimetric nature, and the capacity for use on dried bloodspots make this method more suitable for field use than the Hirono method.

Currently two chromatographic test are licensed and commercially available: BinaxNow^®^ G6PD test and CareStart^®^ G6PD deficiency screening test, the latter introduced more recently. The BinaxNow^®^ G6PD deficiency screening test is a qualitative enzyme chromatographic test which in a laboratory setting distinguished accurately between samples with G6PD activity less than 4.0 U/g of haemoglobin and those with greater enzyme activity [47]. The test needs to be performed between 18°C and 25°C, requires venous blood, a cold chain for the reagents and pipettes, which are a major operational limitation in many settings. The test has been cleared for distribution by the FDA and is used by the US military, but pricing (estimated around USD $25/test) may be prohibitive for national malaria programmes. The CareStart^®^ G6PD deficiency screening test is a qualitative enzyme chromatographic test based on the reduction of light yellowish tetrazolium dye to purple-coloured formazan. The version of the test used in published studies aims to distinguish samples with G6PD activity less than 2.7 U/g of haemoglobin from those with greater enzyme activity [48]. The CareStart^®^ G6PD deficiency screening test is considered promising but, like the BinaxNow product, the currently commercially released version of the test needs to be optimized and validated for use in field settings. Upgrades of the CareStart^®^ G6PD deficiency screening test are in development including a version which allows quantitative estimate of G6PD activity using a reader.

These biochemical approaches offer a functional analysis of G6PD deficiency, but are often difficult to interpret, especially for mosaic female heterozygotes, who may also haemolyse following treatment with 8-aminoquinolines. The interpretation of results of these tests should further take into account the duration of malaria infection, anaemia of the patient and other haematologic parameters. It is possible that an acute febrile episode drives haemolysis. Measured G6PD levels may be reduced during acute malaria episodes. Serial evaluations of G6PD activity during the acute malaria episode and following recovery are on-going in Thailand (see below) and will help define the dynamics of this process. A major challenge for these tests is to ensure their sensitivity in detecting vulnerable populations, choosing an appropriate cut-off value for deficiency and ensuring affordable scale-up in field settings. Neither genetic nor gross biochemical approaches capture the full picture of G6PD activity and hence a combination of assays may be needed for a more complete understanding of G6PD deficiency. Treatment decisions in contrast may be based on rapid diagnostic tests, which are currently in development and will be of critical importance for the safe deployment of primaquine and other 8-aminoquinoline anti-malarials.

### Experiences and perspectives in several Asian Pacific countries on tests to diagnose G6PD deficiency

Testing along the Thai-Burmese border around Mae Sot showed a prevalence of G6PD phenotypic deficiency between 9% and 18% among healthy men of Karen and Burmese ethnicity, using the fluorescent spot test [18,30]. Approximately 90% of deficient subjects identified were of the G6PDd-Mahidol genotype [30]. As part of on-going studies to determine the relationship between G6PD genotype and the risk for primaquine-induced haemolysis, a panel of mutations other than the G6PD-Mahidol genotype will be sequenced. Diagnosing of G6PD deficiency in women using rapid tests carries more uncertainty than in men. This observation combined with the potential dangers of using primaquine in pregnancy could have implications for the population coverage if primaquine mass administrations are to be safely deployed. A better understanding of the risk of haemolysis may be gained through quantitative assessment of G6PD activity using spectrophotometric and cytochemical assays.

Pailin, Cambodia is considered an epicentre of the emergence of multidrug resistant *P. falciparum* and as such a crucial location for deploying primaquine safely as part of strategies aimed at containing resistant parasites [4,53,54]. Primaquine has been used in Cambodia in the past as part of malaria control efforts. There are anecdotal reports of blackwater fever and some deaths. In addition, children presented to one paediatric referral hospital with blackwater fever following the ingestion of traditional medicines, sulphonamides and anti-malarial drugs [55]. Mass treatment has been used in Cambodia, which included a 9 mg dose of primaquine [56]. Although there were no reports of blackwater fever there was no active surveillance for haemolysis or other adverse events. A programme of mass treatment with primaquine in the 1980s (in which the dose of primaquine was not specified) is thought to have resulted in cases of severe and fatal haemolysis, precipitating the complete abandonment of the drug in malaria control. The prevalence of G6PD deficiency in Cambodia is estimated to be between 13% and 26% among males and 3% to 4% among females using diagnostic tests which could have missed heterozygous females [57-59]. The most frequently encountered genotypes are G6PD Viangchan followed by Mahidol, Union, and Coimbra. Recent research conducted in Pailin, in the north-western part of the country, suggests a 15% prevalence of G6PD deficiency among males and 7% among females [48]. According to the WHO classification, 1% of those tested were in class I, 6% in class II and 11% in class III. In 95% of the individuals tested the genotype was G6PD-Viangchan. Experience with the florescent spot test in Cambodia suggests that the test fails to detect reliably heterozygous females. A recent systematic evaluation of the CareStart^®^ G6PD test in this region, suggests a role for this test in the region [48].

In the Philippines routine screening of newborns for G6PD deficiency using direct tests is part of the national newborn screening programme. Espino and co-workers recently evaluated two assays in Puerto Princesa City, Palawan. The direct fluorescent spot test was compared to the rapid colorimetric test WST-8 in their ability to detect G6PD deficiency in 537 high school students. The investigators found that both assays were difficult to implement in field conditions and were unsuitable for screening in the Philippines. In addition the investigators found discrepancies between test results based on venous compared to capillary blood. The discrepancies may be due to higher haemoglobin levels in capillary blood.

A range of surveys in the Indonesia suggests a prevalence of genotypic G6PD deficiency between 0.6% and 14% [38,45,60-70]. The prevalence of genotypes responsible for G6PD deficiency in Indonesia is highly heterogeneous with at least 12 documented G6PD polymorphisms, including Gaohe (95 A > G), Vanua Lava (383 T > C), Mahidol (487 G > A), Mediterranean (563 C > T), Coimbra (592 C > T), Bajo Maumere (844 G > T), Viangchan (871 G > A), Chatham (1,003 G > A), Chinese–5 (1,024 C > T), Surabaya (1,291 G > A), Canton (1,376 C > G), and Kaiping (1,388 G > A). On-going research on Sumba Island by Satyagraha and co-workers found a prevalence of G6PD deficiency ranging between 3.0% and 6.6% in three districts. The overall prevalence of G6PD deficiency in Sumba (Southwest, West and Central) was 4.9%. These prevalence rates mirror the prevailing pattern of malaria endemicity. Preliminary findings suggest a relatively low sensitivity of the CareStart^®^ G6PD deficiency screening test in the field setting in Sumba. Possible explanations include the high humidity, high temperature, and an unstable supply of electricity, all of which rendered the quantitative G6PD gold standard subject to likely error.

Relatively little is known regarding G6PD deficiency in the Solomon Islands [71]. The WST8/1-methoxy PMS method was successfully used by Kuwahata and co-workers as part of a large survey in the Solomon Islands [46] in which 8,541 people from 41 villages were screened in the Isabel province. The prevalence of G6PD deficiency, defined by enzyme activity <30% relative to a wild-type control, was 20% with 7% of individuals classified as having of severe G6PD deficiency (WHO class I-II). The applicability of a filter-paper, mass-screening assay based on the WST8/1-methoxy PMS method to provide prevalence of G6PD deficiency in Isabel Province was evaluated. This study showed that it was possible to undertake large-scale mass screening of G6PD deficiency in the field using the filter-paper assay in order to assess prevalence of the deficiency. The filter-paper method showed complete concordance with biochemical testing. Although these studies demonstrated utility of the test, reliability of G6PD measurements was limited by high humidity and a lack of correction for the storage period.

### Economic perspectives on G6PD testing

There is a growing awareness that robust and reliable G6PD tests that can be used in remote, malaria-endemic regions are needed. The primary demand for G6PD tests comes from malaria control programmes in vivax-endemic countries, where prolonged and higher doses of primaquine are required for radical cure. A single low-dose primaquine (0.25 mg base/kg) can be safely administered even in individuals with G6PD deficiency and is sufficient to kill gametocytes as mentioned earlier [2,3].

Besides the malaria control programmes, the pharmaceutical industry is interested in reliable, affordable point-of-care tests for G6PDd to license and safely roll-out new 8-aminoquinolines. Tafenoquine, the putative successor of primaquine, has been in development for over three decades [72]. Being another 8-aminoquinoline, the potential of tafenoquine to trigger haemolysis in G6PD-deficient people has been the major focus of early Phase I and II studies. Safe deployment of novel 8-aminoquinolines, such as tafenoquine, for the radical cure of vivax malaria will require use of a rapid diagnostic test prior to the administration of the drug in an individual who has not been tested previously. If a specific G6PDd test were to be co-developed as a “companion diagnostic” it would require approval from the relevant regulatory agencies for use with that drug. Alternatively, assuring the availability of a test prior to prescription of the drug without massive waste of unused tests is a complex task. For example, if the diagnostic is supplied separately of the drug physicians will depend on continuous supply of this diagnostic test, requiring a secure but separate supply chain. Since primaquine is currently prescribed without prior testing, introducing a test will have to change physician behaviour. Adding a diagnostic test will also increase the cost of the radical cure of vivax malaria.

Equally important for the development and regulatory approval of rapid diagnostic tests to prevent haemolysis may well be the unresolved question of which level of enzyme activity carries an unacceptable risk for severe haemolysis. The absence of a consensus over what represents an unacceptable level of haemolysis is not only the major challenge for the development of tests to diagnose G6PD deficiency but also a critical barrier to the regulatory approval of candidate 8-aminoquinolines.

Several institutions and companies are currently involved in developing new generation G6PD testing kits. The Program for Appropriate Technology in Health (PATH), an international, non-profit global health organization, has identified the development of diagnostic tests to detect G6PD deficiency as an urgent priority. Other institutions include the Foundation for Innovative Diagnostics (FIND) as well as the UK Department for International Development (DFID) and the Bill and Melinda Gates Foundation (BMGF) have indicated interest in supporting the development of diagnostics to detect G6PD deficiency.

A first step toward the development of a robust clinical test is the description of a target product profile (TPP) of the end product (Table 2). Major challenges identified already include the dependence of G6PD activity on environmental temperature and humidity. Test results should be adjusted by a temperature and perhaps a humidity correction factor. Secondly the correct read-out of reaction rates may be time sensitive. Hence a handheld reader of G6PD tests which could also adjust for environmental temperature may be required to provide accurate and reproducible test results.

**Table 2:** An outline for a possible TPP for a rapid test to detect G6PD deficiency used prior to the treatment of vivax malaria

**Feature**	**Ideal specification**	**Comments**
Output	Level of G6PD deficiency	In the absence of a consensus a quantitative read out may be most appropriate. In the absence of a consensus a qualitative read out may be controversial.
Use case	On patients with confirmed malaria infection.	This is an additional cost to malaria case management.
User	Someone who performs malaria RDTs or microscopy.	A quantitative read-out could come from a reader or scanner.
Sensitivity and Specificity	Must accurately classify all patients with G6PD levels below a pre-defined cut-off. No patients with potentially dangerously low G6PD levels should be misclassified as normal.	It is unclear on what the suitable cut-off should be. Too low and the risk of haemolysis increases. Too high primaquine is denied to more patients who need it.
Environmental tolerance	25-38°C, 40-90% humidity	Enzyme activity is extremely temperature sensitive.
Result read window	< 20 minutes	Technically challenging for an enzyme reaction.
Specimen type	Finger stick	Currently very limited data with finger stick specimens.

### The broader benefits of G6PD testing

The broader benefits of a reliable point-of-care test are likely to be considerable, but benefits to individuals and to society need to be weighed against the potential costs and risks of the overall objective. Such an analysis should consider the alternatives to the introduction of testing, including the status quo (in most cases no primaquine and no testing), as well as the introduction of different primaquine treatment regimens without prior G6PD testing. For the latter, the potential benefits of testing in terms of decreased risk of primaquine-induced haemolysis needs to be balanced against the cost of diagnosis and the potential negative consequences of introducing the tests, including those due to poor test accuracy. The potential impact of rolling out primaquine on endemic parasite populations has not been measured, but may be rationally presumed to be very significant given its broad therapeutic range. Reliable, accurate, affordable and easy to use G6PD testing may dramatically improve primaquine therapy by permitting relatively high dose and short duration regimens rather than the current standard regimen of 14 days’ duration. Ultimately, at the societal level the accumulated costs and consequences of continued malaria transmission, morbidity and mortality need be considered in a global economic analysis.

### Conclusions, research priorities and an action plan

The exploration of safe but efficacious doses of 8-aminoquinolines for the radical cure of vivax malaria and the reduction of falciparum transmission has a high priority in global malaria research (see also Table 3 below).

**Table 3:** Key research priorities

1.	Exploration of safe yet efficacious doses of 8-aminoquinolines for the radical cure of vivax malaria and the reduction of falciparum transmission.
2.	Definition of the relationship between genotype, enzyme activity and co-factors.
3.	Understanding of the relationship between 8-aminoquinoline dose and risk of haemolysis in G6PD normal and deficient individuals.
4.	Determining the correlation between enzyme activity and the severity of haemolysis.
5.	Definition of a threshold of G6PD activity that stakeholders, including regulatory agencies consider sufficient to administer safely standard 8-aminoquinoline regimens.
6.	Consensus on the degree of haemolysis (i e, proportion of red cell lysis) that constitutes an unacceptable clinical risk to the patient.
7.	Investigation of the mean level of haemolysis in uncomplicated malaria without primaquine treatment.
8.	What are acceptable test characteristics (e g, sensitivity and specificity) of rapid tests in various populations and field conditions?
9.	High resolution mapping of G6PD deficiency and haemolysis risks across major malaria endemic settings.
10.	Analysis of the cost-effectiveness of G6PD deficiency tests and the risk benefit of deploying or withholding primaquine regimens for *P. vivax* infections.

The most relevant phenotype regarding potential primaquine sensitivity is residual G6PD enzyme activity but the evidence that informs any correlation between G6PD and primaquine sensitivity phenotypes remains crude. The first step towards a better understanding is the collection of data on the reduction in enzyme activity that results in a significant risk of haemolysis following administration of a standard regimen of an 8-aminoquinoline. A major challenge for G6PD testing is heterozygous females. Lyonization of the trait renders expression of the mutant gene anywhere between 0% and 100% and is a random event. This can result in unrecognized heterozygous females being at risk for haemolysis. This problem requires careful study, applying cytochemical characterization of the erythrocyte populations of girls and women. There is no consensus on what level of enzymatic activity is required to completely exclude severe haemolysis. The current WHO classification suggests a rather arbitrary cut-off of less than 10% of enzyme activity is indicative of a significant risk of severe haemolysis. Neither is there agreement on what constitutes a clinically significant fall in a patient’s haemoglobin concentration. To understand the fraction of the drop in haemoglobin attributable to 8-aminoquinoline therapy it would be critical to understand the level of haemolysis attributable to uncomplicated malaria without 8-aminoquinoline treatment. For a broader understanding between the genotype, the phenotype, the threshold for haemolysis and what represents an unacceptable risk, clinical observations need to be combined with sequence-based analysis of the G6PD gene, cytofluometric assay and candidate diagnostic tests.

The uncertainties regarding the regional distribution of G6PD genotypes suggest a need for phenotypic and genotypic surveys in areas with sparse or outdated data as well as correlates with haemolysis phenotypes. It is not sufficient to conduct such surveillance in selected, established study centres. Specifically, understudied malaria-endemic regions require collection of high-quality data on G6PD deficiency. Current plans to conduct multicentre studies of 8-aminiquinolines could serve as a platform for the in-depth evaluation of G6PD diagnostic kits.

The 8-aminoquinoline doses required to kill the metabolically inactive hypnozoites for the radical cure of vivax malaria are multi-fold higher than doses required to kill gametocytes for the interruption of falciparum malaria transmission [2]. A series of studies conducted in China suggests that low doses of primaquine (0.25 mg base/kg) kill all stages of P.falciparum gametocytes [3]. Expert opinions suggest that such a single, low-dose primaquine (0.25 mg base/kg) can be safely administered even without prior G6PD testing [1]. Once evidence accumulates that this strategy can indeed safely block the transmission of *P. falciparum* the focus of G6PD tests will be the detection of individuals at risk for haemolysis following 8-aminoquinoline therapy targeting hypnozoites.

These strategies target a critically important global health problem: the inability to safely treat a potentially life-threatening infection. The consequences of morbidity and opportunities for the transmission caused by the relapses of vivax malaria have been operationally and scientifically neglected. A better understanding of the risk benefit ratio of deploying or withholding primaquine regimens is crucial for the wider use of 8-aminoquinolines. The Asian nations carrying the bulk of the global *P. vivax* burden now look to the scientific community to finally solve the 60-year-old primaquine toxicity problem and provide a more effective chemotherapeutic management of the many species, stages, and clinical manifestations known collectively as the endemic malarias [73,74].

## Competing interests

The authors declare that they have no competing interests.

## Authors’ contributions

LvS and RP organised the workshop. LvS wrote the first draft of the manuscript. All authors contributed to the content, read and approved the final manuscript.
